# The Dental Protective Association

**Published:** 1890-05

**Authors:** W. H. Dorrance, W. D. Saunders, W. D. Douglass, Wm. Cleland

**Affiliations:** Committee.; Committee.; Committee.; Sec'y, Mich. Dental Ass'n.


### Editorial, Etc.

### THE DENTAL PROTECTIVE ASSOCIATION.

At meeting of the Michigan Dental Association, the principal points in Dr. Crouse's remarks were: First, that by combining in some such an organization as the Dental Protective Association we band the strength of ten thousand men into one—all defence, necessary can be made with but little more expense than would be required for an individual. The preparation of one case will answer for all; all evidence can be collected better by an association than in any other way.

Second, that it is very important that all get into the Association at this time for the reason that an appealed suit to the Supreme Court before the formation of the Association will probably be decided in favor of the Crown Company, owing to deficiency in evidence and imperfect presentation. If the Crown Company win this suit, they will send notices all over the United States, thereby demoralizing the profession and causing many to pay them money which should be in the Protective Association and thus avoid that calamity.

The Protective Association will be ready with new evidence and a new record, and can take care of its members against any claims of the Crown Company. If you are not in the Association, the Association will not take care of you when the time comes. If each man in the profession will pay $10 and assume a responsibility of ten more, without any farther assessments, we will have an organization that is sure to break up all this abuse and save the dental profession an annual outlay of on an average of $100 each to unjust claimants.

Remember, it will cost more than $10 later to join and will certainly cost more than that if you do not protect yourselves.

*Resolved*, That the Michigan Dental Association heartily approves the aim and plan of the Dental Protective Association and that it is further

*Resolved*, That it is the duty of every member of the dental profession in this State to join the Dental Protective Association.

*It is also resolved* that Dr Crouse be requested to furnish the dental journals with an abstract of his remarks for publication.

W. H. DORRANCE,
W. D. SAUNDERS,
—— DOUGLASS,
Committee.

WM. CLELAND, *Sec'y*,
Mich. Dental Ass'n.

